# Whole-Exome Sequencing Identifies Novel *SCN1A* and *CACNB4* Genes Mutations in the Cohort of Saudi Patients With Epilepsy

**DOI:** 10.3389/fped.2022.919996

**Published:** 2022-06-22

**Authors:** Muhammad Imran Naseer, Angham Abdulrhman Abdulkareem, Mahmood Rasool, Hussein Algahtani, Osama Yousef Muthaffar, Peter Natesan Pushparaj

**Affiliations:** ^1^Center of Excellence in Genomic Medicine Research, King Abdulaziz University, Jeddah, Saudi Arabia; ^2^Department of Medical Laboratory Technology, Faculty of Applied Medical Sciences, King Abdulaziz University, Jeddah, Saudi Arabia; ^3^Department of Biochemistry, Faculty of Science, King Abdulaziz University, Jeddah, Saudi Arabia; ^4^King Abdulaziz Medical City, King Saud Bin Abdulaziz University for Health Sciences, Jeddah, Saudi Arabia; ^5^Department of Pediatrics, Faculty of Medicine, King Abdulaziz University, Jeddah, Saudi Arabia

**Keywords:** Epilepsy, *SCN1A*, *CACNB4*, WES, Saudi patients

## Abstract

Epilepsy is a neurological disorder described as recurrent seizures mild to severe convulsions along with conscious loss. There are many different genetic anomalies or non-genetic conditions that affect the brain and cause epilepsy. The exact cause of epilepsy is unknown so far. In this study, whole-exome sequencing showed a family having novel missense variant c.1603C>T, p. Arg535Cys in exon 10 of Sodium Voltage-Gated Channel Alpha Subunit 1 (SCN1A) gene. Moreover, targeted Sanger sequencing analysis showed c.1212A>G p.Val404Ile in *SCN1A* gene in 10 unrelated patients and a mutation in Calcium Voltage-Gated Channel Auxiliary Subunit Beta 4 gene where one base pair insertion of “G” c.78_79insG, p.Asp27Glyfs^*^26 in the exon 3 in three different patients were observed from the cohort of 25 epileptic sporadic cases. The insertion changes the amino acid sequence leading to a frameshift mutation. Here, we have described, for the first time, three novel mutations that may be associated with epilepsy in the Saudi population. The study not only help us to identify the exact cause of genetic variations causing epilepsy whereas but it would also eventually enable us to establish a database to provide a foundation for understanding the critical genomic regions to control epilepsy in Saudi patients.

## Introduction

Generalized epilepsy with febrile seizures plus (GEFS+; MIM#604233) is a condition of an epileptic syndrome involving the multiple genes, including febrile seizures (FS), febrile seizures plus (FS+) along with partial and generalized seizures, such as myoclonic-astatic epilepsy (MAE) and myoclonic epilepsies of infancy (SMEI) (1, 2).

The *SCN1A* is the clinically most relevant epilepsy gene that is involved in the coding of the voltage-gated Na+ channel alpha subunit NaV1.1, *SCN1A* is a seizure disorder that is carried out as an autosomal dominant manner or the patient may have a *de novo* variant leading to the pathogenic conditions. The percentage of the disease due to the *de novo* pathogenic variants differs by phenotypic conditions (3). This gene is located on 2q24.3 and it is composed of 26 exons with 6,030 base pairs. *SCN1A* is mainly expressed in the central nervous system (CNS) and in cardiac myocytes known to control the neuronal excitability and expressed in the central and peripheral nervous (4). The mean function of this protein is to maintain the sodium ions' flow into the cells. NaV1.1 channels are responsible for transmitting signals from one nerve cell to another. Through the neurotransmitters released from one neuron and transferred to the next neurons. The NaV1.1 channels control the flow of sodium ions and determine when neurotransmitters need to release.

The proportion of proband with an *SCN1A* gene linked seizure disorder is decreased in affected parents and in the proband severity of the disease increases causing mostly *SCN1A* gene-related severe myoclonic epilepsy in infancy (SCN1A SMEI), intractable childhood epilepsy with generalized tonic-clonic seizures (ICE-GTC) and Dravet syndrome (DS) that may be the result of a pathogenic *de novo* mutation. The proband with an *SCN1A* seizure disorder has almost 50% pathogenic variant from parents. Moreover, the risk of development of seizures is <100% because of decreased penetrance (3). Several studies show that the point mutations in the *SCN1A* gene or deletions on chromosome 2q24.3 have been observed that are causing generalized epilepsy (GE) with febrile seizures plus (GEFS+), DS, or SMEI in the patients (5).

Nowadays, 900 distinct mutations are reported for the *SCN1A* gene. These mutations often result in of with seizure disorders. Most of the time the *de novo* variation of the *SCN1A* gene has been linked in about 80% of patients with DS. Moreover, ICE-GTC and GEFS+ in the form of missense type were first described by (1, 6). Approximately 90% were missense mutations and only 10% were either frameshift or truncation mutations (7).

The *CACNB4* is a very important gene that is involved in the control of calcium channels encoding β4 is mainly expressed in the brain and an increased expression level was observed in the early development (8–10). *CACNB4* gene experiences a wide range of alternative splicing and the result showed different variants (β4a, β4b, β4c, and β4e) with distinct subcellular localizations and functions (11–13).

Previously, a missense mutation p.Arg468Gln was reported that was inherited from his father in the *CACNB4* gene having febrile seizures. A detailed and electrophysiological examination of the heterologous expression system showed that after the mutation p.Arg468Gln in *CACNB4* showed increased Ba^2+^ current density as compared to the wild type and this increased Ca_v_2.1 currents density increased the neurotransmitter release in the excitatory neurons underneath the disorder of insufficient inhibitory neurons (14). It was also reported that a homozygous mutation c.377T>C where p. (Leu126Pro) in the *CACNB4* gene is pathogenic that is also linked with the reported disease phenotype.

In humans, various neurological phenotypes are linked with heterozygous mutation reported in *CACNB4* (MIM: 601949), such as a female patient, having the juvenile myoclonic epilepsy (JME) was reported with nonsense mutation c.1444C>T leading to the frameshift where the protein p.Arg482^*^ (MIM: 607682). Moreover, another family has two affected members with rare generalized tonic-clonic seizures and idiopathic generalized epilepsy (IGE) having non-synonymous variants c.311G>T and p.Cys104Phe in *CACNB4*, episodic ataxia (MIM: 613855) was reported in a family in which five members were suffered from disease along with two members showed the p. (Cys104Phe) variant were normal (15). The presence of the heterozygous c.1403G>A, p.Arg468Gln *CACNB4* variant has been proposed to create more neurological disorders in those patients having a pathogenic *SCN1A* mutation where the current densities increased in calcium channel (14). Epilepsy and ataxia were also reported due to the heterozygous mutation in *CACNB4A* (15), whereas the Cacnb4 knockout mice showed severe neurological phenotype (16–18).

In this study, whole-exome sequencing (WES) for one family and targeted sequencing was done for 25 samples having epilepsy and our results showed that the family had novel missense variant c.1603C>T, p. Arg535Cys in exon 10 of *SCN1A* gene and c.1212A>G p.Val404Ile in 10 unrelated patients along with a mutation in *CACNB4* gene in exon 3 c.78_79insG, p.Asp27Glyfs^*^26 in three different patients from the cohort.

## Materials and Methods

### Ethical Approval and Sample Collections

The ethical approval number (013-CEGMR-02-ETH) for this study was taken from the local ethical committee of the Center of Excellence in Genomic Medicine Research, King Abdulaziz University Jeddah. The sample collection and experimental work were done according to the international guidelines mentioned in the Declaration of Helsinki 2013. Blood from the patients recruited for this study was collected in the center after having the informed written consent signed by the patient or guardians in case children were involved in the study. DNA was extracted from blood stored in the EDTA tubes (Roche Life Science), as previously described (19) until further use for DNA extraction. The concentration of DNA was measured by using Nanodrop^TM^ 2000/2000c spectrophotometers (Thermo Fisher Scientific Waltham, Massachusetts, USA).

### Clinical Report of the Patient

Patients of different ages and sex male and female recruited for this study had different types of epileptic syndromes involving the multiple genes, namely, GEFS+, FS, and FS+ with generalized and partial seizures along with MAE and myoclonic epilepsies of infancy (SMEI). Some of the patients had the phenotype of intractable childhood epilepsy with generalized tonic-clonic seizures and Dravet syndrome. The phenotype in the recruited patients was also showing minor variation even among the same family members with the same pathogenic variant. While overall all the patients had a similar phenotype as in the case of previously defined conditions of Generalized epilepsy with febrile seizures plus (GEFS+; MIM#604233) in the literature.

### Whole-Exome Sequencing

To find the cause of epilepsy with the gene of potential variants in the enrolled family WES was done using Illumina NextSeq 550 (High-Output v2 kit). The products were sequenced on an Illumina NextSeq instrument with 2 × 76 paired-end reads as previously described (20, 21). The high quality was obtained by maintaining the quality control check on Illumina sequencing platforms. DNA library templates were constructed with accurate quantitation. The standard curve was generated by Roche's Rapid library standard quantification solution and calculator using fluorescence readings and calculating the library sample concentration.

All exomes were considered for sequence gain > 95% of bases covered for at least 15 reads throughout the whole exome. After WES, the FASTQ files were converted to BAM files, which were then converted to variant call format (vcf) files with total variants obtained. Further variants obtained were used for the identification of mutations causing the disease based on rare/novel (MAF+0.01%) frequency, homozygous or heterozygous state functional (predicted damage by Polyphen/SIFT), pathogenicity, genomic position, and protein damaging effect and linkage with the disease and its phenotype. Bioinformatics tools were used by applying various filters. GRCh37 database was used for the alignment of the reference sequence. The obtained variants were further filtered to find out the disease linked with the identified mutation in available public databases, such as for allele frequencies <5.0% in the Genome Aggregation Database (gnomAD, http://gnomad.broadinstitute.org/), and non-sense, frameshift, and splice-site variants in disease-associated genes with a minor allele frequency ≤1.0%, were observed in gnomAD. However, variants with minor allele frequencies ≤5.0% in gnomAD in a patient-specific phenotype-driven gene. The evidence for phenotype causality was then evaluated for each variant resulting from the filtering strategies above, and variants were classified based on the American College of Medical Genetics and American College of Pathologists (ACMG/AMP) criteria (22). ClinGen rule specifications (https://www.clinicalgenome.org/working-groups/sequence-variant-interpretation/). Variants were reported according to HGVS nomenclature (https://varnomen.hgvs.org/). Moreover, *in silico* studies were done for missense variants to predict the effect of amino acid substitutions on protein structure. Identified candidate variant was evaluated using two online software programs: SIFT (http://swissmodel.expasy.org) and PolyPhen-2 software (http://genetics.bwh.harvard.edu/pph2). We followed the standard guidelines of the ACMG. Human gene mutations (database of variant annotations published in the literature). Deleterious effects and abnormalities were also identified using *in silico* analysis for the structure and function of the identified variant leading to the disease. Different software were used, such as PhyloP (https://www.ncbi.nlm.nih.gov/pmc/articles/ PMC4702902/), 1000 Genomes database (http://www.internationalgenome.org/), and PhyloP. SIFT (http://sift.bii.a-star.edu.sg/), GERP++ (http://mendel.stanford.edu/SidowLab/downloads/gerp/ downloads/gerp/), PhastCons (http://compgen.cshl.edu/phast/), CADD (https://cadd.gs.washington.edu/), SiPhy (https://portals.broadinstitute.org/genome_bio/siphy/index.html), and Exome Aggregation Consortium (http://exac.broadinstitute.org/) for the analysis of the identified variants.

### Sanger Sequencing

Sanger sequencing was done for the targeted sequencing of *SCN1A* and *CACNB4* genes and also to validate the obtained result after WES as shown in Supplementary Tables 1, 2 for *SCN1A* and *CACNB4* genes primers sequence. For the Sanger sequencing analysis primers for the exons were synthesized using the primer 3 program to identify the mutations. After the PCR, 1st and 2nd purifications were done by following the protocol. A list of the sequencing primers used for the study was added in Supplementary Tables 1, 2. Sequencing data files were obtained from the AB1 sequencing unit. The sequencing file was aligned with the reference sequence using the BioEdit software. Unrelated healthy samples from the population were used as control samples. We followed the guidelines to search for variations in the National Center for Biotechnology Information (NCBI) SNP database.

### Mutation Analysis

The effects of substituting cysteine for arginine at the amino acid position 535 and isoleucine for valine at amino acid position 404 on the SCN1A protein (PDB ID: 7DTD) and alpha fold model (AF-P35498-F1) were performed using missense 3D software. The secondary structure change, cavity change, buried/exposed change, allowed phi/psi, disulfide bond break, buried charge change, collision, buried hydrophilic residue insertion, buried salt bridge break, buried charge insertion, buried glycine replacement, buried charge replacement, buried proline insertion, buried H-bond break, and glycine in a bend were studied to evaluate the structural impact of these two mutations in the SCN1A protein (23, 24). Furthermore, the Protein Variation Effect Analyzer (PROVEAN) tool (25, 26) that predicts the substitution or indel of an amino acid in a protein structure impacts the biological function was used to evaluate the substitution of isoleucine for valine at amino acid position 404 and cysteine for arginine at the amino acid position in SCN1A protein. For evolutionary conservation, PROVEAN and MAPP software were used to identify the structure/function of the protein. The homology model of the CACNB4 protein was constructed using the automated homology modeling platform Swiss Model (27). The FASTA sequence of *CACNB4* was downloaded from the UniProt Knowledgebase.

## Results

### Whole-Exome Sequencing

**Figure 1:**
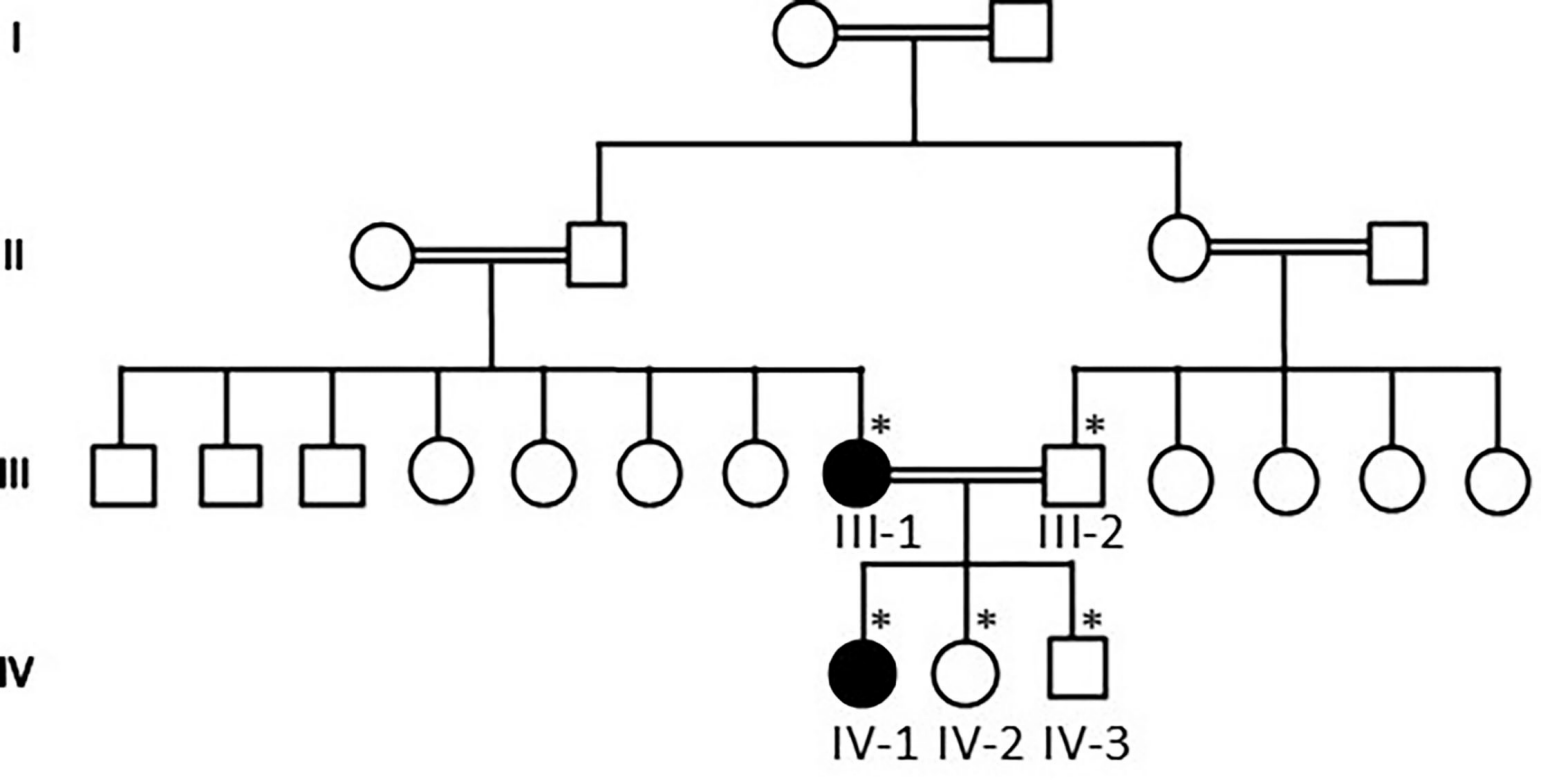
Pedigree of the family showing that consanguineous marriages and III-1 mother and IV-1 are the affected members of the family. The * sign represents the samples available for the study.

**Figure 2:**
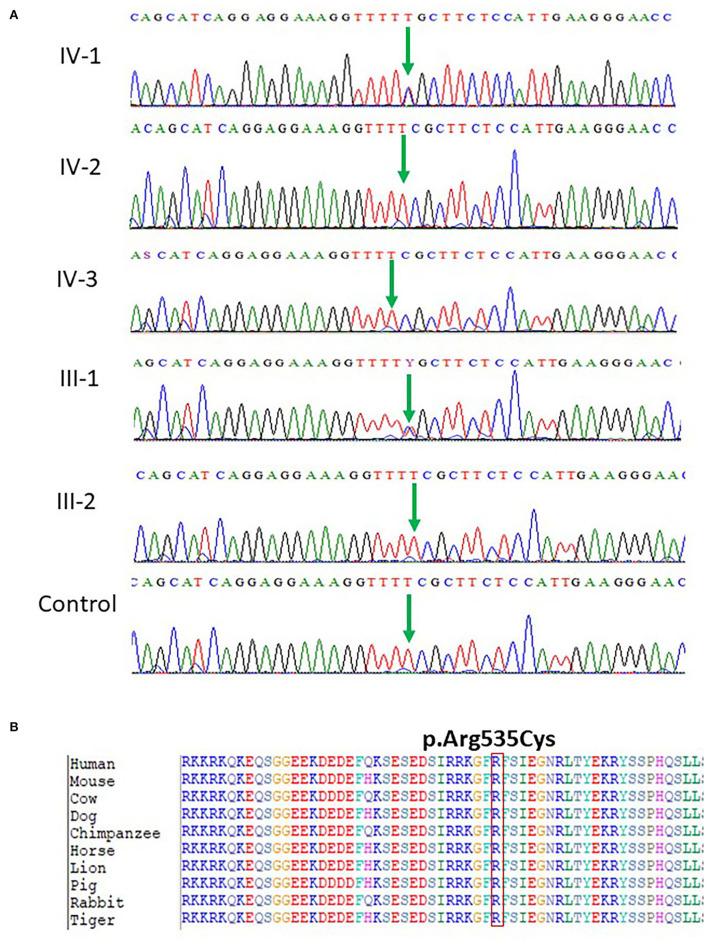
**(A)** Chromatogram of novel missense variant c.1603C>T, p.Arg535Cys in exon 10 of the *SCN1A* gene showing that III-1 (mother) and IV-1 (affected girl) are heterozygous having C/T alleles, while all other members are normal having T/T alleles. **(B)** Protein alignment of different species was done to show the highly conserved amino acid variants p.Arg535Cys *SCN1A* gene highlighted in all species.

Our WES results identified novel missense variants c.1603C>T and p.Arg535Cys in exon 10 of the *SCN1A* gene with an autosomal dominant inheritance in an affected family where the mother III-2 and her daughter IV-1 were affected with GEFS+ and obtained pedigree showed that two members were affected as shown in Figure 1. Both the affected III-1 (mother) and IV-1 (affected girl) were heterozygous having C/T alleles, while all other members were normal having T/T alleles as shown in Figure 2A.

The identified missense mutation c.1603C>T and p.R535C in the *SCN1A* gene (NM_001165963.1) a pathogenic variant identified in Saudi patients with epilepsy has not been reported previously. The change of amino acid from arginine to cysteine showed a big physicochemical difference that is expected to change the impact of secondary protein structure due to polarity difference, size, charge, and many other properties. The high missense variants' Z-score of 5.61 was shown by the *SCN1A* gene suggesting a low rate of benign missense variation. So far, 335 pathogenic missense variant has been identified in the *SCN1A* gene leading to a common mechanism of disease. The missense variant p.R535C is disease-causing as predicted by both PolyPhen2 and SIFT. While the c.1603C>T nucleotide in *SCN1A* is predicted conserved by PhyloP and GERP++ among 100 vertebrates as shown in Figure 2B.

**Figure 3:**
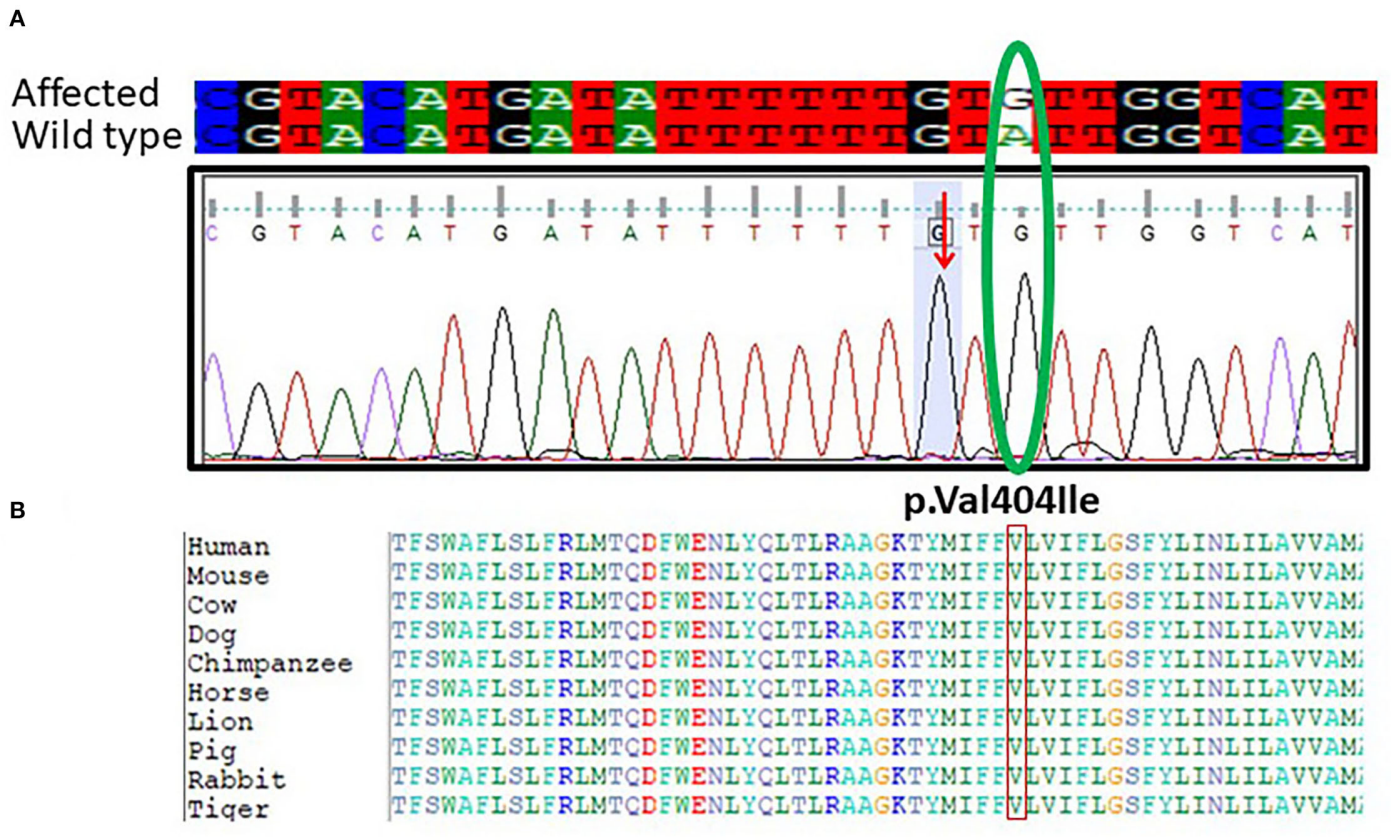
**(A)** Chromatogram of Sanger sequencing showing the c.1212A>G p.Val404Ile in 10 unrelated patients *SCN1A* gene from the cohort of 25 epileptic sporadic patients. The green oval is showing the change in the base pair in mutant and while type sequence. **(B)** Protein alignment of different species was done to show the highly conserved amino acid variants p.Val404Ile *SCN1A* gene highlighted in all species.

Furthermore, targeted Sanger sequencing for *SCN1A* and *CACNB4* genes was also done. We identified variants in the *SCN1A* gene in 10 epileptic patients with febrile seizure and (GEFS+) from mild to moderate DS along with intractable childhood epilepsy. The patients showed a mutation in exon 9 c.1212A>G and p.Val404Ile in *SCN1A* positioned at 166903445 on chromosome 02 as shown in Figure 3A. The p.Val1404Ile variant is novel (not in any individuals) in 1 kG. There is a small physicochemical difference between valine and isoleucine, which is not likely to impact secondary protein structure as these residues share similar properties. The missense variant p.Val404Ile might be a disease-causing as predicted by SIFT. While the c.1212A>G nucleotide in *SCN1A* is predicted conserved by PhyloP and GERP++ among most of the vertebrates Figure 3B.

**Figure 4:**
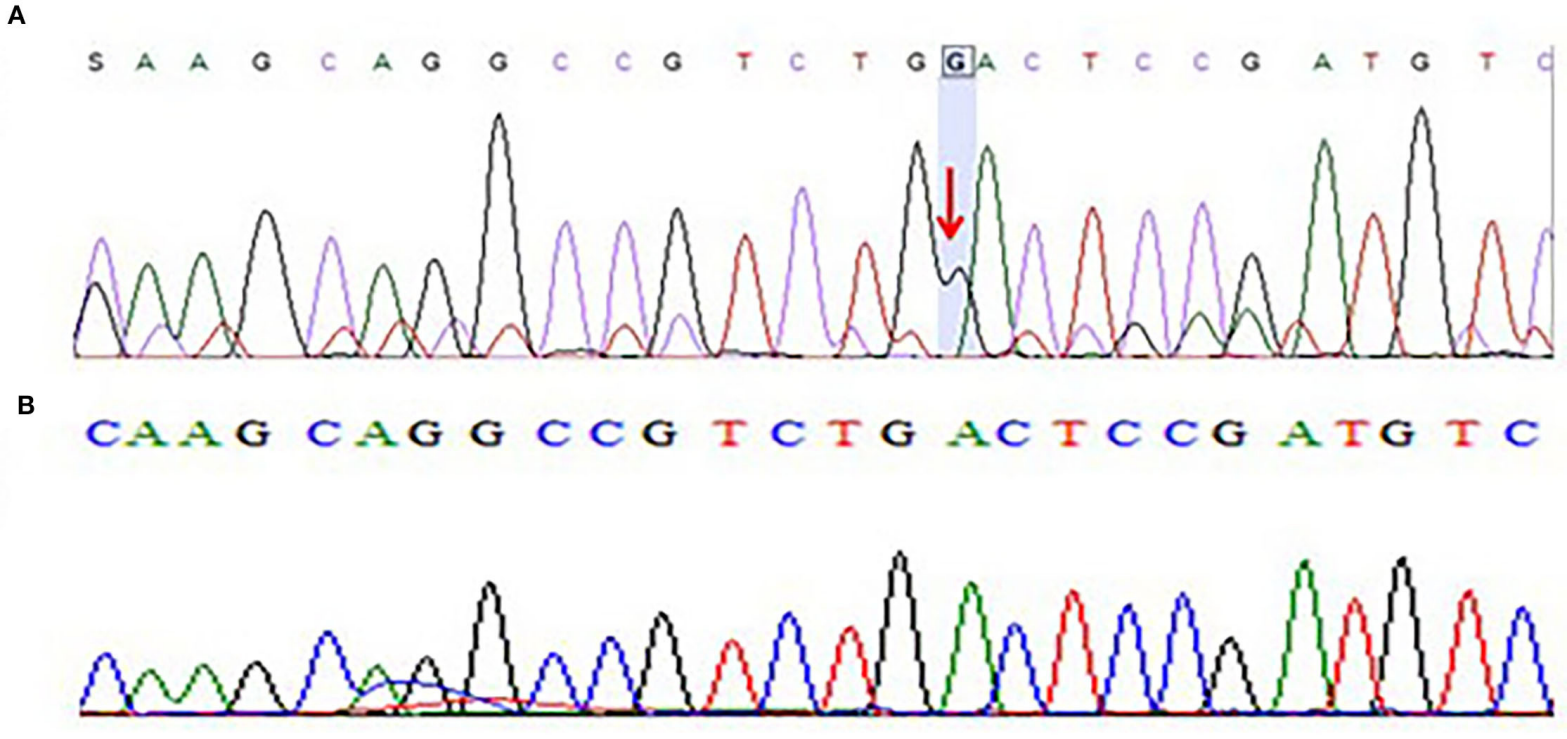
**(A)** Chromatogram of Sanger sequencing showing one base pair insertion of “G” nucleotide in exon 3 of *CACNB4* gene sequence of c.78_79insG, p.Asp27Glyfs*26 in three different patients from the cohort of 25 epileptic sporadic patients leading to a frameshift mutation. **(B)** Chromatogram of Sanger sequencing showing wild-type sequence of the *CACNB4* gene (obtained from 100 healthy individuals as the control group).

Moreover, we have also sequenced the coding regions of the *CACNB4* gene. The results of sequencing analysis showed one base pair (G) insertion in exon 3 of the *CACNB4* gene in 3 patients. The mutation was positioned at 15279851-15279852 on chromosome 02 where c.78_79insG in the *CACNB4* gene. This insertion leads to changes in the amino acid sequence and results in a frameshift mutation that may change the protein structure in the affected members as shown in Figure 4.

### Sanger Sequencing

After the WES, the obtained results of novel mutations were further validated using Sanger sequencing analysis after designing the targeted forward and reverse primers for the *SCN1A* and *CACNB4* genes. The results of Sanger sequencing analysis confirmed the WES novel missense variant c.1603C>T, p. Arg535Cys in exon 10 of *SCN1A* gene in one family and c.1212A>G p.Val404Ile in 10 unrelated patients from the cohort of 25 epileptic sporadic patients as shown in Figures 2A, 3A. Moreover, sequence analysis showed an insertion of the “G” base pair in the exon 3 sequence of c.78_79insG, p.Asp27Glyfs^*^26 in the *CACNB4* gene in three different unrelated patients as shown in Figure 4A. The identified novel mutations were also sequenced in 100 normal control from the population Figure 4B.

### Mutation Analysis of SCN1A

**Figure 5:**
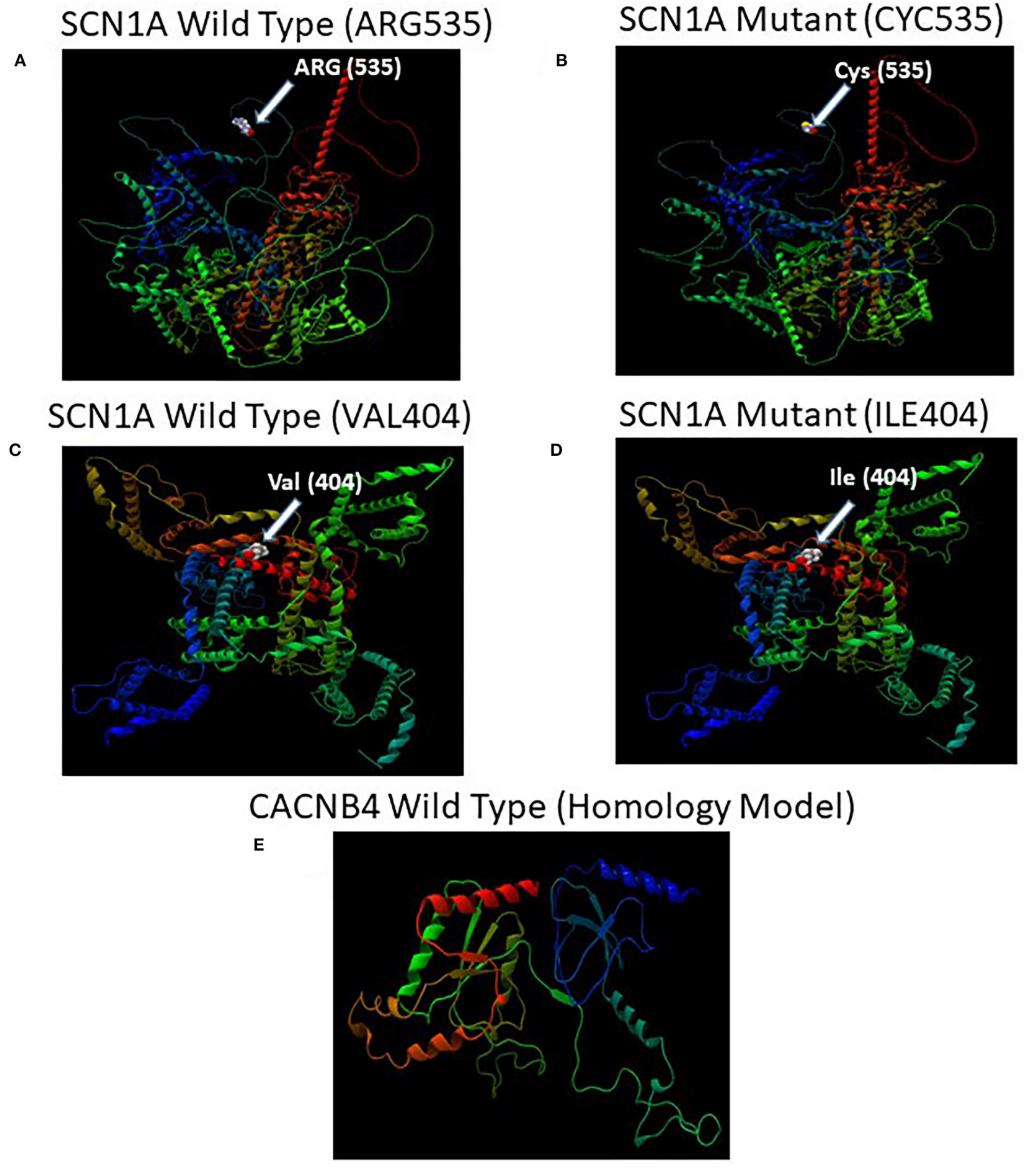
**(A)** Illustration of the predicted wild-type and mutant structures (AF-P35498-F1) of *SCN1A* and the prediction for the position of p.Arg535Cys. **(B)** The amino acid Arg at position 535 in the wild-type *SCN1A* and Cys at position 535 in the mutant *SCN1A* are shown as space-filling [(Calotte or Corey, Pauling, and Koltun (CPK)] models. **(C)** Illustration of the wild-type and mutant structures (PDB ID: 7DTD) of *SCN1A* and the prediction for the position of p.Val404Ile. **(D)** The amino acid Val at position 404 in the wild-type *SCN1A* and Ile at position 404 in the mutant *SCN1A* are shown as space-filling [(Calotte or Corey, Pauling, and Koltun (CPK)] models. **(E)** The 3D structure of the wild-type *CACNB4* structure was designed using the SWISS-MODEL homology modeling platform (27).

The missense 3D analysis of the substitution of cysteine by arginine at the amino acid position 535 in SCN1A protein (Alphafold model) revealed no structural damage as shown in Figures 5A,B. Moreover, the missense 3D analysis revealed no structural damage in SCN1A protein (EM structure) due to the replacement of valine by isoleucine at the amino acid position 404 as shown in Figures 5C,D. However, the protein variation effect analysis using PROVEAN predicted (cut-off −2.5) that R535C mutation was deleterious with a PROVEAN score of −4.772 and V404I mutation in SCN1A was neutral with a PROVEAN score of −0.816. For evolutionary conservation, PROVEAN and MAPP software were used to identify the structure/function of the protein. The homology model of the CACNB4 protein was constructed using the automated homology modeling platform Swiss Model as shown in Figure 5E.

## Discussion

In this study, we are reporting a Saudi family with a novel missense variant in exon 10 c.1603C>T, p. Arg535Cys and 10 sporadic cases also showed mutation c.1212A>G p.Val404Ile in *SCN1A* gene from the cohort of 25 epileptic sporadic patients. In the same cohort of epileptic patients, three of them also showed mutation as insertion of one base pair (G) in exon 3 sequence of c.78_79insG, p.Asp27Glyfs^*^26n in the *CACNB4* gene, and this insertion caused a frameshift mutation leading to the change in protein structure causing disease.

Voltage-dependent sodium channels are complexes that are involved in the control of sodium interchange between extracellular and intracellular spaces that are very important to control action potentials in the brain (neurons) and in muscle cells. At chromosome 2q24 cluster of sodium channel, including *SCN1A, SCN2A*, and *SCN3A* genes, are encoded. The *SCN1A* gene is considered the most important and relevant gene responsible for epilepsy for encoding the α-1 subunit of voltage-gated sodium channel mainly expressed in the brain (28). Any mutation in the *SCN1A* gene causes mild to severe type of epilepsies known as DS that occur due to *de novo* mutation first time in a person with epilepsy with no family history. The phenotypes of the *SCN1A* gene depend upon the location of the mutations. In most cases, *de novo* mutations are reported in SMEI along with missense, truncation, and deletions of large exons (29, 30). Phenotype and genotype associations identified that missense mutations in *SCN1A* in the important useful area were mostly linked to SMEI, in rare families that were considered to have the severe phenotype of GEFS+ (31–33).

Families with negative history of the disease cannot be ruled out unless proper evaluations was done. In most cases, families have *SCN1A*, GEFS+ linked with the parents, while the negative family history may be due to failure to recognize the disease or low penetrance, or early death before the onset of disease symptoms. Moreover, if the disease occurs the first time that may be due to the somatic mosaicism for the pathogenic variant affected only mildly or minimally (34). In most cases, the *de novo* pathogenic variant is the cause of *SCN1A* Dravet syndrome and intractable childhood epilepsy with ICE-GTC (35). Very less around 5% of patients having DS may have the same *SCN1A* pathogenic variant in parents as well (31, 36, 37). In the database of *SCN1A* gene mutations (http://www.caae.org.cn/gzneurosci/scn1adatabase/data), 1,528 mutations are linked with epilepsy out of a total of 1,727 reported mutations (38). Of the mutations related to epilepsy among them, 945 are linked to severe SMEI, while 263 are associated with severe myoclonic epilepsy (SME) and 18 are associated with partial epilepsy (PE), whereas 151 are linked with severe myoclonic epilepsy borderline (SMEB), 31 are related to partial epilepsy and febrile seizures plus (PEFS+), 8 are related to GE, moreover, 55 are related to GEFS + (38).

The *CACNB4* gene protein coded for the locus plays a very important function in controlling the calcium channel through G protein inhibition, increased calcium current, regulatory the alpha-1 subunit membrane targeting along with fluctuating the voltage requirement for activation and inactivation. Some reported mutations in the *CACNB4* gene are linked with JME, IGE, and episodic ataxia, type 5. In previous studies, it has been reported that the *CACNB4* gene is associated with neurological disorders. Episodic ataxia and JME are due to the heterozygous mutation in the form of missense variant p.Cys104Phe and nonsense variant p.Arg482^*^ in *CACNB4* leading to the disease and it was a rare mutation that was found non-Finnish European only in a single individual (15). Episodic ataxia was also reported in a cohort of patients having a pathogenic mutation in the *CACNB4* (39). Calcium channels are very important and help to maintain the contact and provide the controlled release of neurotransmitters in the nerve cell in the brain through which signals from one neuron to another are passed that further support the neurons' survival.

In a recent study, heterozygous *CACNB4* mutations are not linked with epilepsy (40). The heterozygous parents of the two affected members having p.Leu126Pro variation were normal. Likewise, the heterozygous mutated animal model did not show any type of deformities (41). Remarkable pathogenicity prediction scores were high for the change p.Leu126Pro, conservation of leucine 126 was high and this variant was not present in databases whereas, the mutation on *CACNB4* in both alleles causes severe neurological conditions in the two affected members of the family (42).

In this study, next-generation sequencing (NGS) technology was used to identify the mutations in the cohort of epilepsy families instead of using Sanger sequencing analysis for each gene linked with epilepsy. The latest genomic technique improving the rate and reducing the costs has cut down the time and the expenses of diagnostic tests greatly especially linked with genetic diagnosis of epilepsy and it has simultaneously boosted its analytical sensitivity. Clinical variability and genetic locus heterogeneity are critical for many diseases. Since it is money and time consuming to test genes and locus one by one with Sanger sequencing, NGS is the best technology to solve these problems (43–46). NGS allows genes of the same or similar diseases to be tested together in one gene panel. This conversion in gene testing enhances the analytical sensitivity of the test (47, 48). This is especially significant for diseases that are caused by both *de novo* or Mendelian mutations, such as epilepsy-related voltage-gated sodium channel mutations. For patients who are undiagnosed after a few traditional approaches, displaying extreme heterogeneity and having ambiguous phenotype, exome sequencing, and whole-genome sequencing provide a more effective way of discovering disease-related genes. Epilepsy is a complex neurological disorder so to identify the exact genetic cause we used this technique and identified two novel missense mutations in the *SCN1A* gene along with frameshift mutation in *CACNB4*. This study provides all possible genetic variant in epilepsy and related to disease diagnosis and prognosis and further, enable us to provide a foundation for understanding the critical genomic regions that will help to solve the epileptic problem in Saudi patients.

## Conclusion

In the cohort of epilepsy samples, we identified a family with novel missense variant c.1603C>T, p. Arg535Cys in exon 10 of the *SCN1A* gene. Whereas, the targeted Sanger sequencing showed c.1212A>G p.Val404Ile mutation in 10 sporadic samples from the Saudi population. Moreover, in this study, we find insertion in exon 3 of the *CACNB4* gene leading to a frameshift mutation in c.78_79insG, p.Asp27Glyfs^*^26 in three different patients. The study will help to present a better description of the genetic variations in epilepsy and would eventually enable us to identify not only the diagnosis and prognosis but also the correct treatment approach. Furthermore, it will also help to establish a foundation for collecting data on critical genomic regions which might be involved in the development of an epilepsy database and control of the disease in Saudi patients.

## Data Availability Statement

The datasets for this article are not publicly available because family consent to publicly share the data was not allowed. Requests to access the datasets were directed to mimrannaseer@yahoo.com.

## Ethics Statement

The studies involving human participants were reviewed and approved by the Ethical Committee of Center of Excellence in Genomic Medicine Research CEGMR, King Abdulaziz University Jeddah. Written informed consent to participate in this study was provided by the participants' legal guardian/next of kin.

## Author Contributions

MN, AA, and MR designed the experiments. MN and AA conducted the experiments. PP, OM, HA, and MR analyzed the data. MN, PP, MR, and AA wrote the manuscript. PP, OM, AA, and MN finally revised the manuscript. All authors contributed to the editing of the manuscript and the scientific discussions.

## Funding

This research work was funded by Institutional Fund Project under Grant No (IFPRC-013-247-2020).

## Conflict of Interest

The authors declare that the research was conducted in the absence of any commercial or financial relationships that could be construed as a potential conflict of interest.

## Publisher's Note

All claims expressed in this article are solely those of the authors and do not necessarily represent those of their affiliated organizations, or those of the publisher, the editors and the reviewers. Any product that may be evaluated in this article, or claim that may be made by its manufacturer, is not guaranteed or endorsed by the publisher.

## Supplementary Material

Supplementary Table 1 Showing the list of primers for the *SCN1A* gene used for Sanger sequencing.

Supplementary Table 2 Showing the list of primers for the *CACNB4* gene used for Sanger sequencing.

## Abbreviations

ACMG/AMP: The American College of Medical Genetics and American College of Pathologists
CNS: Central nervous system
vcf: Variant call format
OMIM: Online Mendelian Inheritance in Man
WES: Whole-exome sequencing
SCN1A: Sodium Voltage-Gated Channel Alpha Subunit 1
CACNB4: Calcium Voltage-Gated Channel Auxiliary Subunit Beta 4
GEFS+: Generalized epilepsy with febrile seizures plus
FS: Febrile seizures
FS+: Febrile seizures plus
MAE: Myoclonic astatic epilepsy
SMEI: Myoclonic epilepsies of infancy
SME: Severe myoclonic epilepsy
PE: Partial epilepsy
SMEB: Severe myoclonic epilepsy borderline
PEFS +: Febrile seizures plus
JME: Juvenile myoclonic epilepsy
NGS: Next-generation sequencing
IGE: Idiopathic generalized epilepsy
DS: Dravet Syndrome
ICE-GTC: Intractable childhood epilepsy with generalized tonic-clonic seizures.
