# Supplementary material for: Whole-Exome Sequencing Identifies Novel SCN1A and CACNB4 Genes Mutations in the Cohort of Saudi Patients With Epilepsy
Source: Front Pediatr. 2022 Jun 22;10:919996. doi: 10.3389/fped.2022.919996 (PMC9257097; doi:10.3389/fped.2022.919996)
Supplement: Supplementary Table 1 — Showing the list of primers for the SCN1A gene used for Sanger sequencing. [file Table_1.DOCX]

**Supplementary Table 1:** Showing the primer sequencing used for the *SCN1A* gene sequencing.

| Exons | Sequence |
| --- | --- |
| SCN1AE1F: | 5’- CCTCTAGCTCATGTTTCATGACA-3’ |
| SCN1AE1R: | 5’- TGCATAAACCACCCACAAATGT-3’ |
|  |  |
| SCN1AE2F: | 5’- GTTTGTTTTCATGGGGCACT-3’ |
| SCN1AE2R: | 5’- TTTTCATCACTCTCATTTTGCA-3’ |
|  |  |
| SCN1AE3F: | 5’- ATAACACTTGGATTTTGATTTTCT -3’ |
| SCN1AE3R: | 5’- GGAATGACTTTAATAAAATGCTCT -3’ |
|  |  |
| SCN1AE4F: | 5’- TTCCACTGATGGAGTGATAAGAAA-3’ |
| SCN1AE4R: | 5’- TTTGGTTTTCCATCAGTGCTT-3’ |
|  |  |
| SCN1AE5F: | 5’- GGCTCTTTGTACCTACAGCTTTTT-3’ |
| SCN1AE5R: | 5’- GCATCCTTCTCTGCTTGACA-3’ |
|  |  |
| SCN1AE6F: | 5’- CTCCACTAGCGTTGCAAACA-3’ |
| SCN1AE6R: | 5’- ACTTGAGGGGCTGGATATCC-3’ |
|  |  |
| SCN1AE7F: | 5’- AACCTGACCTTCCTGTTCTCA-3’ |
| SCN1AE7R: | 5’- **AGAAACTGAAGATAACAGCCAACT**-3’ |
|  |  |
| SCN1AE8F: | 5’- AAATCTTTCTGACTAGGCAATATCA-3’ |
| SCN1AE8R: | 5’- **CAAGAAAACCAATTGAGTTATGAA**-3’ |
|  |  |
| SCN1AE9F: | 5’- TTGAAAGTTGAAGCCACCAC-3’ |
| SCN1AE9R: | 5’- GAGCAGGTGGTTGTATGAGGA-3’ |
|  |  |
| SCN1AE10F: | 5’- AAGCCATGCAAATACTTCAGC-3’ |
| SCN1AE10R: | 5’- TAAAACATGGGAGAGAGGGG-3’ |
|  |  |
| SCN1AE11F: | 5’- TGCAAAATGAAATCACATTCAA-3’ |
| SCN1AE11R: | 5’- GAACGGCAACCTTGAGAATG-3’ |
|  |  |
| SCN1AE12F: | 5’- GTCACCATTTGGTTGTTTGC-3’ |
| SCN1AE12R: | 5’- TTGTGAGTTGGGAATAGTGCA-3’ |
|  |  |
| SCN1AE13F: | 5’- AGTGGATATGAAATAAATGTGTGTG-3’ |
| SCN1AE13R: | 5’- TCAAATTTTCTGCTTGAAAAACTG-3’ |
|  |  |
| SCN1AE14F: | 5’- GAATCATTGTGGGAAAATAGCA-3’ |
| SCN1AE14R: | 5’- TTTGCCACACAACCATTAGC-3’ |
|  |  |
| SCN1AE15F: | 5’-TGACCATTTCTAGGTAAAGCTCAAT-3’ |
| SCN1AE15R: | 5’-AAATACACCAGCATGGCACA-3’ |
|  |  |
| SCN1AE16F: | 5’-CTGCTCTTCCCTACATTGGTG-3’ |
| SCN1AE16R: | 5’-CATATACACACATACAGATGATCCTCA-3’ |
|  |  |
| SCN1AE17F:1 | 5’-AAAAGGGTTAGCACAGACAATGA-3’ |
| SCN1AE17R:1 | 5’-TGTTGGTCAGCTAAAATAAATCACA-3’ |
|  |  |
|  |  |
| SCN1AE18F: | 5’-AAAGGATATTATGGAAGCAGAGACA-3’ |
| SCN1AE18R: | 5’-TGACTTATTAATCCTTTGTTTCATTG-3’ |
|  |  |
| SCN1AE19F: | 5’- CTGCCCTCCTATTCCAATGA-3’ |
| SCN1AE19R | 5’- TCAGACCACTATGTTATGTTGACAGA-3’ |
|  |  |
| SCN1AE20F:1 | 5’- CACATTGAATGATGATTCTGTTTAT-3’ |
| SCN1AE20R:1 | 5’- **GCAAAAGGAATGGCAAATTC**-3’ |
|  |  |
| SCN1AE21F: | 5’- TTTTGAACCTTGCTTTTACATATCC-3’ |
| SCN1AE21R: | 5’- AGTTTATGAGCCCAGATGGG-3’ |
|  |  |
| SCN1AE22F: | 5’- TGTCTTTCAAAAATATTCCCTTTTG-3’ |
| SCN1AE22R: | 5’- CGAATAAAGCATAAACGACCAA-3’ |
|  |  |
| SCN1AE23F: | 5’- CACCAGTGACATTTCCAGCA-3’ |
| SCN1AE23R: | 5’- AGTGTTTTCTCTGCCAAATGAG-3’ |
|  |  |
| SCN1AE24F: | 5’- TTTTTAGCCATCCATTTTCTATTTT-3’ |
| SCN1AE24R: | 5’- AAAAAGAAAGTAGCTAACATTTCCA -3’ |
|  |  |
| SCN1AE25F:1 | 5’- CAAAAATCAGGGCCAATGAC-3’ |
| SCN1AE25R:1 | 5’- **CAAGATCATCCCAGCAATCA**-3’ |
|  |  |
| SCN1AE26F:1 | 5’- **TCTGAACCTTACCTTGGAGCTT**-3’ |
| SCN1AE26R:1 | 5’- **CATCATCATATCCTTCCTGGTT**-3’ |
|  |  |
| SCN1AE26F:2 | 5’- **GCATGATCTGCCTATTCCAAA**-3’ |
| SCN1AE26R:2 | 5’- **ACAAAGCGGGTTCTAGGAGA**-3’ |
|  |  |
| SCN1AE26F:3 | 5’- **CAGCTCATTGCCATGGATTT**-3’ |
| SCN1AE26R:3 | 5’- **AGGAGGTCAATGCCAAACTG**-3’ |
