# Supplementary material for: Whole-Exome Sequencing Identifies Novel SCN1A and CACNB4 Genes Mutations in the Cohort of Saudi Patients With Epilepsy
Source: Front Pediatr. 2022 Jun 22;10:919996. doi: 10.3389/fped.2022.919996 (PMC9257097; doi:10.3389/fped.2022.919996)
Supplement: Supplementary Table 2 — Showing the list of primers for the CACNB4 gene used for Sanger sequencing. [file Table_2.DOCX]

| Exons | Sequence |
| --- | --- |
|  |  |
| CACNB4-1F | 5’- CTTTAAGGTGCCGCGCTCT-3’ |
| CACNB4-1R | 5’- CTTCCTCACACCTCCTCCG-3’ |
|  |  |
| CACNB4-2F | 5’- GAGTCGCGGAGGAGACTTG-3’ |
| CACNB4-2R | 5’- CAGTCCCTCTCCCTCTAGCC-3’ |
|  |  |
| CACNB4-3F | 5’- CTCGCCCCAGGATACTGTTA-3’ |
| CACNB4-3R | 5’- GCTACCAGCTGCTCTTCCCT-3’ |
|  |  |
| CACNB4-4F | 5’- CACTACCTGGTACTATCAGAGTTTCAA-3’ |
| CACNB4-4R | 5’- TGAGCCAAACTAGCCAGAGAG-3’ |
|  |  |
| CACNB4-5F | 5’- GCATTTTATTCTTCTTGTCCCCTA-3’ |
| CACNB4-5R | 5’- ATTTTCAAGGGTGTATACTTTCAAA-3’ |
|  |  |
| CACNB4-6F | 5’- TAGGATGAGGGCCAAAGAAA-3’ |
| CACNB4-6R | 5’- TTTGAACACACATGCAAGTAGTTTT-3’ |
|  |  |
| CACNB4-7F | 5’-AAAGTGGGCGATAGGAAGGT-3’ |
| CACNB4-7R | 5'-ATTTCAGTGACCCAAGTGGC-3' |
|  |  |
| CACNB4-8F | 5’- ATGAATGTTGTGGCTGTGGA-3’ |
| CACNB4-8R | 5’- CAGTCATGTTGACGCTTGCT-3’ |
|  |  |
| CACNB4-9F | 5’- AGTACCCTTCCCTCCCTCCT-3’ |
| CACNB4-9R | 5’- TGTTTCCAGAGATTTCAGAAGTTC-3’ |
|  |  |
| CACNB4-10F | 5’- CACTTGGAGAGCCCCATAAC-3’ |
| CACNB4-10R | 5’- GCTGACACATGGTGCATTTG-3’ |
|  |  |
| CACNB4-11F | 5’- TGTAATGTAGACCAACATATCTGAAAA-3’ |
| CACNB4-11R | 5’- TGGACAAAGGAGAACGTCTTG-3’ |
|  |  |
| CACNB4-12F | 5’- GGCCAAGCAGACCATTAAAA-3’ |
| CACNB4-12R | 5’- TGATGGTGATGATGGTATGTTG-3’ |
|  |  |
| CACNB4-13F | 5’-TGCCCATATAGCACCTCAAA-3’ |
| CACNB4-13R | 5’-CTTGATTAGGATGGAGACTGGA-3’ |
|  |  |
| CACNB4-14F | 5’- GCCTTATTAAGTCAGATATTTAGCATC-3’ |
| CACNB4-14R | 5’- TTACTGTGCTAATGGGCTGC-3’ |
|  |  |

**Supplementary Table 2:** Showing the primer sequencing used for the *CACNb4* gene sequencing.
